# Analog Least Mean Square Loop for Self-Interference Cancellation: A Practical Perspective †

**DOI:** 10.3390/s20010270

**Published:** 2020-01-03

**Authors:** Anh Tuyen Le, Le Chung Tran, Xiaojing Huang, Yingjie Jay Guo

**Affiliations:** 1 School of Electrical and Data Engineering, University of Technology Sydney, Ultimo 2007, Australia; xiaojing.huang@uts.edu.au (X.H.); jay.guo@uts.edu.au (Y.J.G.); 2 School of Electrical, Computer and Telecommunications Engineering, University of Wollongong, Wollongong 2522, Australia; lctran@uow.edu.au

**Keywords:** in-band full-duplex, self-interference cancellation, adaptive filter, I/Q imbalance, ALMS loop

## Abstract

Self-interference (SI) is the key issue that prevents in-band full-duplex (IBFD) communications from being practical. Analog multi-tap adaptive filter is an efficient structure to cancel SI since it can capture the nonlinear components and noise in the transmitted signal. Analog least mean square (ALMS) loop is a simple adaptive filter that can be implemented by purely analog means to sufficiently mitigate SI. Comprehensive analyses on the behaviors of the ALMS loop have been published in the literature. This paper proposes a practical structure and presents an implementation of the ALMS loop. By employing off-the-shelf components, a prototype of the ALMS loop including two taps is implemented for an IBFD system operating at the carrier frequency of 2.4 GHz. The prototype is firstly evaluated in a single carrier signaling IBFD system with 20 MHz and 50 MHz bandwidths, respectively. Measured results show that the ALMS loop can provide 39 dB and 33 dB of SI cancellation in the radio frequency domain for the two bandwidths, respectively. Furthermore, the impact of the roll-off factor of the pulse shaping filter on the SI cancellation level provided by the prototype is presented. Finally, the experiment with multicarrier signaling shows that the performance of the ALMS loop is the same as that in the single carrier system. These experimental results validate the theoretical analyses presented in our previous publications on the ALMS loop behaviors.

## 1. Introduction

The sub-6 GHz spectrum will be more and more congested as the Third Generation Partnership Project has indicated that the fifth-generation (5G) new radio systems will operate in this frequency band. In-band full-duplex (IBFD) technology that allows transceivers to simultaneously transmit and receive signals in a single frequency band will be extremely useful to enhance spectrum efficiency [1]. IBFD transmission is also beneficial to avoid collision in multiple access networks by solving the problem of hidden terminals. Furthermore, this new transmission technology helps to reduce round-trip latency in multi-hop relay networks since the relay can forward while receiving packets [2]. Finally, IBFD has a great potential in military applications, such as a joint jammer and communication system. In such system, the IBFD transceiver can be an attacker to the opponents, while it is receiving the signal of interest [3].

However, realizing IBFD transceivers is challenging due to the fundamental problem of self-interference (SI). Under the IBFD operation, the strong signal emitted from the transmitter is in the same frequency band and is much stronger than the signal of interest. Consequently, SI saturates the receiver and blocks it from receiving the desired signal from the far-end. Therefore, great efforts have been devoted to mitigate the SI to a level below the noise floor of the receiver.

Self-interference can be consecutively mitigated in the propagation, radio frequency (RF), and digital domains [2]. Propagation domain methods aim to attenuate the level of SI by strategically locating transmit and receive antennas [4,5], polarizing transmit and receive antennas [6,7,8,9], or sharing one antenna by a circulator [10,11]. Digital domain approaches generate the cancellation signal by utilizing the baseband transmitted data and channel state information (CSI) to suppress the digitalized residual SI after the analog-to-digital converter (ADC) [5]. Meanwhile, the RF domain approaches aim to generate a cancellation signal by modifying a reference signal to mimic the SI at the input of the receiver. As indicated in [12], RF domain cancellation is the most important step since propagation domain approaches are limited by the device size and digital domain cancellation only works if the ADC is not saturated. Some typical examples of RF cancellers are proposed in [13,14,15,16].

In most of the RF adaptive cancellers, digital signal processing (DSP) and CSI are required to calculate the weighting coefficients and modify the phase and amplitude of the reference signal. The involvement of DSP makes these cancellers more complicated, especially in IBFD multiple-input multiple-output (MIMO) systems [17]. In addition, the need of CSI requires the transceiver to frequently operate in a half-duplex mode to update the channel information. An approach that does not require DSP can be found in [18], where a circulator combined with an adjustable impedance mismatched terminal (IMT) circuit is used to generate the cancellation signal from the reflected SI. However, this method is only suitable for a single antenna system because it may be very challenging to tune the IMT circuit in more complex SI channels, such as those in IBFD MIMO systems. The reason is that, in IBFD MIMO systems, the SI at each receive antenna is a combination of the interference from all transmit antennas [17]. The analog least mean square (ALMS) loop proposed in [19] is an advantageous structure due to its simplicity and ability to achieve significant SI cancellation without any DSP and CSI. The promising application of the ALMS loop in IBFD MIMO systems has been presented in [20] with a beam-based analog SI cancellation structure. The behaviors of this adaptive filter are comprehensively investigated in [12,19,21,22,23,24]. It has been shown that the performance of the ALMS loop depends on the transmitted signal properties and the structure of the loop. Particularly, the roll-off factor of the pulse shaping filter in the transmitter, the loop gain, the tap delay and the number of taps in the loop determine the level of cancellation given by the ALMS loop. However, these results are only confirmed by simulations in MATLAB (R2016B), rather than by hardware experiments. In addition, although the ALMS loop has a simple structure, its implementation using off-the-shelf components is still a challenging task. For example, the multipliers with a high conversion gain required in the loop are not available for RF signals.

Therefore, this study focuses on the practical perspective of the ALMS loop. A part of this work has been presented in [25] which shows a prototype of the ALMS loop and some measurement results. In this paper, we firstly review the implementation of various RF multi-tap adaptive filters for SI cancellation published in the literature. Then, a practical structure of the ALMS loop is proposed, and a prototype of the loop is implemented to provide experimental results. Contributions of this paper are summarized as follows:

- A comprehensive review on the implementation of the state-of-the-art adaptive filters for the RF domain cancellation in IBFD radios is presented.
- A practical structure of the ALMS loop for its future applications is proposed. Although adaptive filters employing the least mean square algorithm in the analog domain had been implemented as in [26,27], they are for low frequency (lower than 1 MHz) applications only. Based on the proposed structure, a prototype of the ALMS loop including two taps in an IBFD system at the carrier frequency of 2.4 GHz is implemented.
- Experimental results are provided to validate the theoretical analyses in our previous publications. Measured results show that 39 dB and 33 dB of SI mitigation can be achieved by the prototype in the system with 20 MHz and 50 MHz bandwidths, respectively. From the parameters of the components used in the prototype, the level of cancellation can be verified by the analytical formula of interference suppression ratio provided in [12]. Considering the degradation factor given in [24], the practical results agree with the theoretical ones. The level of cancellation is also measured with different roll-off factors of the pulse shaping filter to confirm the analyses shown in [22]. Finally, the prototype is evaluated with a 20 MHz-bandwidth orthogonal frequency-division multiplexing (OFDM) signal to confirm that the ALMS loop works well in both single carrier and multicarrier signaling systems as mentioned in [21,22,24].

The remainder of this paper is organized as follows. Section 2 reviews some related works. In Section 3, the practical structure of the ALMS loop is proposed, and the implementation of the prototype using off-the-shelf components is described. In Section 4, experimental results are presented. Finally, Section 5 concludes the paper.

## 2. Related Works

### 2.1. Analog Multi-Tap Adaptive Filters

**Figure 1:**
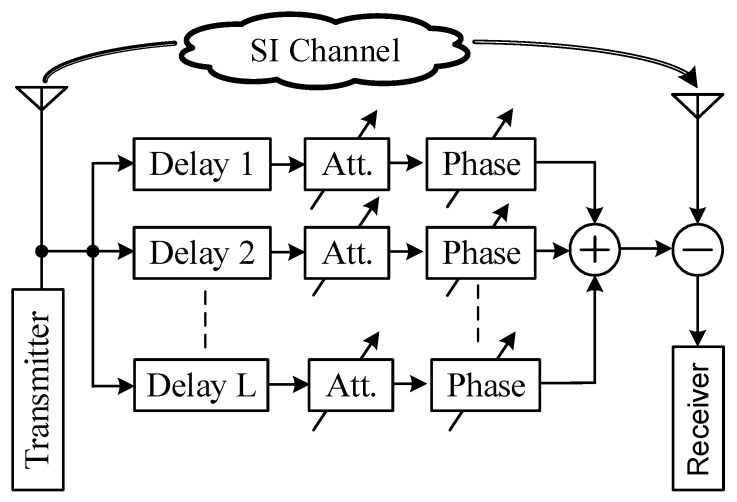
Multi-tap adaptive filter structure.

As indicated in the introduction, the RF domain cancellation is an essential step to mitigate the SI. The authors in [1] pointed out that, among different approaches in the RF domain cancellation, multi-tap adaptive filters are able to cancel the transmit noise and nonlinear distortion components since they utilize the transmitted signal to generate the cancellation signal. A generalized structure of this approach is depicted in Figure 1. Since the SI channel includes multi-paths, this approach aims to mimic the SI channel by a multi-tap structure. Each tap includes a delay line and a mechanism to independently modify the amplitude (by an attenuator) and phase (by a phase shifter) of the delayed reference signal. The outputs of all the taps are combined together before canceling the SI at the input of the receiver.

Although analog multi-tap adaptive filters are similar in principle, they are implemented in different ways, especially in the mechanism to tune the amplitude and phase of the reference signal. Some prototypes of analog multi-tap adaptive filters are compared in Table 1. In this comparison, we focus on the implementation aspect of these structures. The performance aspect represented by the interference suppression ratio (ISR) is for reference only since these prototypes are evaluated and designed for IBFD systems with different transmission bandwidths at different transmit powers.

It can be seen that most of these cancellers [11,14,16] require DSP performed by field programmable gate arrays (FPGA) to synthesize the weighting coefficients for the tuning mechanism. The authors in [13] employed down-converters to calculate the weighting coefficients in the baseband by an intergrator. In addition, the delay line can be implemented by either integrated circuits (IC) [13], microstrip traces [11,16], or coaxial cables [14]. One advantage of the IC delay line is that it can provide a long delay time comparable to microstrip traces and coaxial cables in a compact size. It is also worth noting that, with only two taps, the level of RF domain cancellation can reach 33 dB in a 20 MHz IBFD system as in [13].

### 2.2. ALMS Loop Architecture

**Figure 2:**
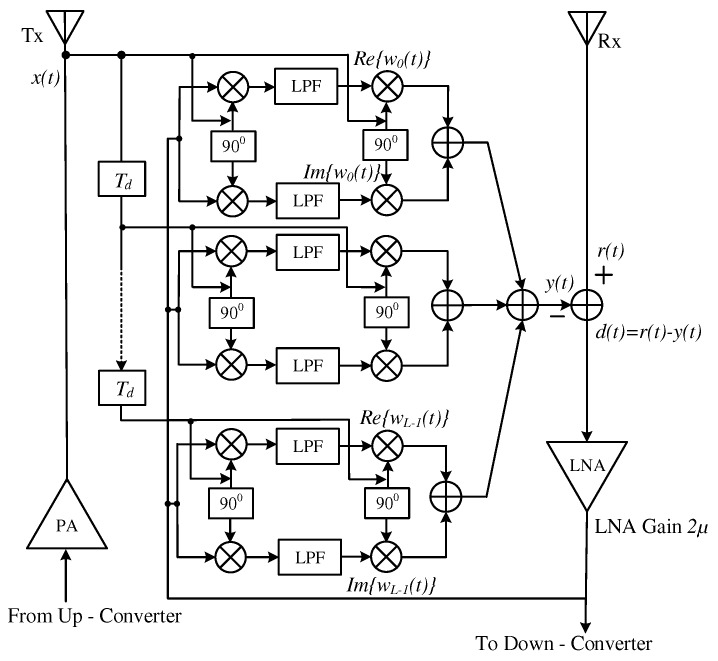
The ALMS loop structure.

The ALMS loop proposed in [19] is a multi-tap canceller without any DSP involvement. Instead, the tuning mechanism in each tap is carried out by two simple resistor-capacitor (RC) low-pass filters (LPF) and two quadrature multiplier pairs. The architecture of the ALMS loop is depicted in Figure 2. The delay line $T_{d}$ aims to create a delayed version of the reference signal to mimic the multipath components in the SI signal. At each tap, the amplitude and phase of the delayed reference signal are modified at the second I/Q multiplier pair by the two weighting coefficients which are generated from the first I/Q multiplier pair followed by the respective LPFs. The outputs of all the taps are combined together to generate the cancellation signal y(t), which will be used to cancel the SI in the received signal r(t). The residual signal d(t) is amplified by a low-noise amplifier (LNA) and looped-back to the first I/Q multiplier pair in each tap.

To demonstrate the operation of the ALMS loop, we use the signal models as follows. Assuming that the IBFD radio is a single carrier system, the modulated data symbols $a_{n},n=-\infty ,\ldots ,\infty$, are filtered by the root-raised cosine (RRC) pulse shaping filter g(t) before being up-converted to RF and amplified by the power amplifier. The transmitted signal x(t) at the transmit antenna is modeled as x(t)=Re{X(t)ej2πfct}, where $f_{c}$ is the carrier frequency, and X(t) is the baseband equivalent, which can be described as

(1) $$X\left(t\right)=\sum_{n=-\infty }^{\infty }V_{X}a_{n}g\left(t-nT_{s}\right),$$

where $T_{s}$ is the symbol interval and $V_{X}$ is the root mean square amplitude of the transmitted signal. Note that the pulse shaping filter is assumed to have a unit power, i.e., $\frac{1}{Ts}\int_{0}^{T_{s}}{\left|g\left(t\right)\right|}^{2}dt=1$. In addition, we assume that the transmitted data symbols $a_{n}$ are independent of each other, i.e.,

(2) $$E\left\{a_{n}^{*}a_{n^{\prime }}\right\}=\left\{\begin{array}{cc} \; & 1,\;\mathrm{for}\;n=n^{\prime },\\ \; & 0,\;\mathrm{for}\;n\neq n^{\prime }, \end{array}\right.$$

where E{.} stands for ensemble expectation. The average power of X(t) is defined as $\frac{1}{T_{s}}\int_{0}^{T_{s}}{E\{|X\left(t\right)|}^{2}\}dt=V_{X}^{2}$ over an 1 Ω load.

Due to the IBFD operation, the received signal r(t) includes the remote signal s(t), the SI z(t), and the additive Gaussian noise n(t), i.e., r(t)=s(t)+z(t)+n(t). For theoretical analyses, the SI channel is assumed to be a multi-tap filter so that z(t) can be expressed as

(3) z(t)=Re∑l=0L−1hl*X(t−lTd)ej2πfct,

where $h_{l},l=0,1,\ldots ,L-1,$ are the SI channel coefficients and $T_{d}$ is the delay between adjacent taps. Note that the ALMS loop does not require CSI to synthesize the weighting coefficients in its taps. Therefore, although the SI channel is modeled as a multi-tap filter with the tap delay $T_{d}$, the actual SI channel may have arbitrary path delays. In that case, if $T_{d}<T_{s}$, the model in Equation (3) can still be used to approximate the real SI channel with a modeling error, which has been derived in [19]. The cancellation signal y(t) is combined from the *L* taps of the ALMS loop as

(4) y(t)=Re∑l=0L−1wl*(t)X(t−lTd)ej2πfc(t−lTd),

where $w_{l}\left(t\right)$ is the complex weighting coefficient at the *l*-th tap. The residual signal d(t)=r(t)−y(t) is amplified by the LNA and looped-back to all the taps of the ALMS filter. As shown in [19], the weighting coefficient $w_{l}\left(t\right)$ at output of the LPF in the *l*-th tap is

(5) $$\begin{array}{cc} w_{l}\left(t\right)= & \frac{2\mu \alpha }{K_{1}K_{2}}\int_{0}^{t}e^{-\alpha (t-\tau )}d\left(\tau \right)X\left(\tau -lT_{d}\right)e^{j2\pi f_{c}\left(\tau -lT_{d}\right)}d\tau , \end{array}$$

where α is the decay constant of the RC LPF (α=1/RC), $K_{1}$ and $K_{2}$ are the dimensional constants of the first and second I/Q multiplier pairs, respectively, and 2μ is the gain of the LNA. The dimensional constant $K_{i}$ of a multiplier is determined by its input voltages $v_{i_{1}},v_{i_{2}}$ and its output voltage $v_{o}$ as

(6) $$K_{i}=\frac{v_{i_{1}}v_{i_{2}}}{v_{o}}$$

for i=1,2.

After cancellation, the residual SI signal, denoted as v(t)=z(t)−y(t), can be expressed as

(7) v(t)=Re∑l=0L−1(hl*−wl*(t)e−j2πfclTd)X(t−lTd)ej2πfct.

Denoting $u_{l}\left(t\right)=h_{l}-w_{l}\left(t\right)e^{j2\pi f_{c}lT_{d}}$ as the difference between the channel coefficient and the weighting coefficient in the *l*-th tap of the ALMS loop, we can see that the power of $u_{l}\left(t\right)$ indicates the performance of the ALMS loop. By evaluating $u_{l}\left(t\right)$ in different scenarios, the behaviors of the ALMS loop have been investigated and published in [12,19,21,22,23,24].

Table 2 summarizes some publications on the ALMS loop. In [19], the ALMS loop is firstly introduced and its performance related to the loop gain and the roll-off factor β of the pulse shaping filter is presented through cyclostationary and stationary analyses. Then, the performance of the ALMS loop with different transmitted signal properties is analyzed in [21,22,23]. Furthermore, in [12], the loop gain, roll-off factor, tap delay and number of taps are all considered. It is shown that, when $LT_{d}$ is sufficiently large, the level of cancellation will approach an interference suppression lower bound (ISRLB), defined as the lower bound of the ratio between the residual SI power after cancellation and the SI power without cancellation and given by

(8) $$\begin{array}{cc} ISRLB & =\frac{1+\beta (\sqrt{a+1}-1)}{{\left(1+a\right)}^{2}}, \end{array}$$

where $a=\mu \frac{V_{X}^{2}}{K_{1}K_{2}}\frac{T_{s}}{T_{d}}$ and β is the roll-off factor of the pulse shaping filter. From (8), we see that high-gain multipliers are generally required to achieve the lower ISRLB. Finally, the impacts of in-phase/quadrature (I/Q) imbalances of the quadrature multipliers on the level of cancellation are evaluated in [24]. In particular, a degradation factor of the level of cancellation caused by I/Q imbalances is determined over a range of amplitude and phase errors of the quadrature multipliers.

However, all these findings are only based on theoretical analyses and verified by simulations in MATLAB. A prototype, therefore, is necessary to obtain practical results to validate these theoretical findings. In the next section, we propose a practical structure of the ALMS loop using discrete components.

## 3. Implementation of ALMS Loop

**Figure 3:**
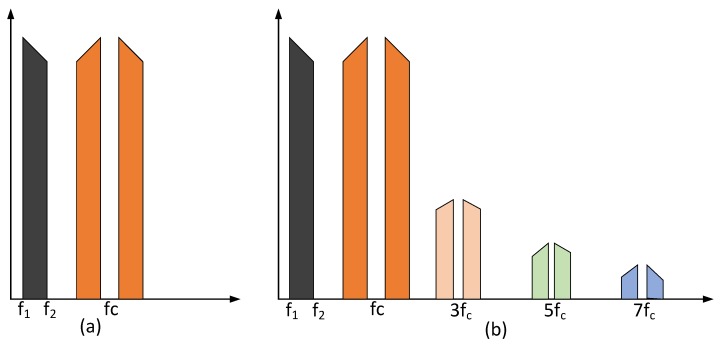
Output spectra of (**a**) a multiplier or a modulator with an LPF and (**b**) an unfiltered modulator [28].

Although the ALMS loop structure is simple, it is still challenging to be implemented using off-the-shelf components. In particular, the high-gain quadrature multipliers in the ALMS loop are unavailable in the RF range. Therefore, we adopt quadrature demodulators and modulators combined with amplifiers to replace the ideal multipliers. However, this replacement faces some new problems. Firstly, unlike an ideal multiplier which can accept any signal in its frequency range, a modulator/demodulator normally requires a single tone with a stable amplitude as a local oscillation (LO) signal. In addition, the multipliers in the ALMS loop are assumed to have a high conversion gain, which is not normally applicable to modulators/demodulators. Therefore, when a modulator and a demodulator are used in the ALMS loop, the reference signal should be provided to their LO ports. Then, a variable gain amplifier is used at these ports to ensure an adequate level of the LO signal. The low conversion gain of the modulator/demodulator can be compensated by an amplifier after combining all the outputs of the taps. Another problem is that, while a multiplier is a linear device, a modulator/demodulator is nonlinear [28]. This means the output of the latter includes some odd harmonics of the carrier frequency as shown in Figure 3b. If an LPF is used following the modulator/demodulator, these harmonics can be removed and the modulator/demodulator can produce the same output spectrum as that of an ideal multiplier as shown in Figure 3a [28].

In the structure of the ALMS loop (cf. Figure 2), the LPFs are not only used to synthesize the weighting coefficients, but also eliminate the odd harmonics at the output of the demodulator. As for the modulator, since its output will be amplified by an amplifier, the harmonics at the output of the modulator will also be attenuated if the cut-off frequency of this amplifier is suitably selected (see the end of this section for more details).

As can be seen in Figure 2, many power splitters are required to split and combine signals. Therefore, Wilkinson dividers are used for both splitters and combiners. The subtractor at the input of the receiver can also be implemented by a Wilkinson divider with a phase shifter, which makes a 180-degree phase shift to the cancellation signal. However, one problem with the Wilkinson divider is that it is a lossy component. Hence, a variable gain amplifier should be used at the input of the RF port of each demodulator to compensate this loss.

**Figure 4:**
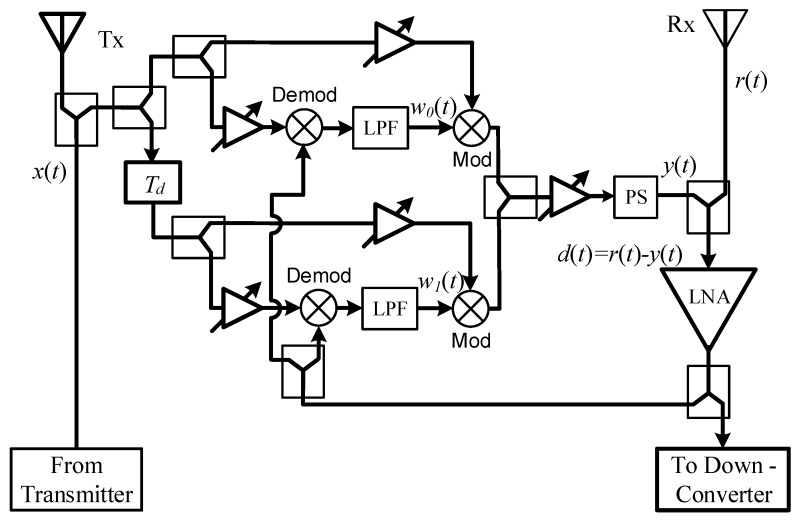
A practical structure of the ALMS loop.

The implementation structure of a 2-tap ALMS loop is presented in Figure 4. Due to the presence of the aforementioned extra components, the loop gain of this structure now can be calculated as

(9) $$G=\mu \frac{V_{LO}^{2}}{K_{1}K_{2}}G_{O},$$

where $V_{LO}$ is the root-mean square amplitude of the reference signal at the LO ports of the modulators and demodulators, and $G_{O}$ is the power gain of the amplifier at the output of the cancellation circuit after compensating the losses of the phase shifter and the power combiner. Therefore, the interference suppression ratio, denoted as ISR, given by the prototype can be expected as

(10) $$\begin{array}{c} ISR\leq \frac{{\left(1+a^{\prime }\right)}^{2}}{1+\beta (\sqrt{a^{\prime }+1}-1)}, \end{array}$$

where $a^{\prime }=G\frac{T_{s}}{T_{d}}$. Note that ISR here is defined as the ratio between the power of the SI without cancellation and that of the residual SI after cancellation.

**Figure 5:**
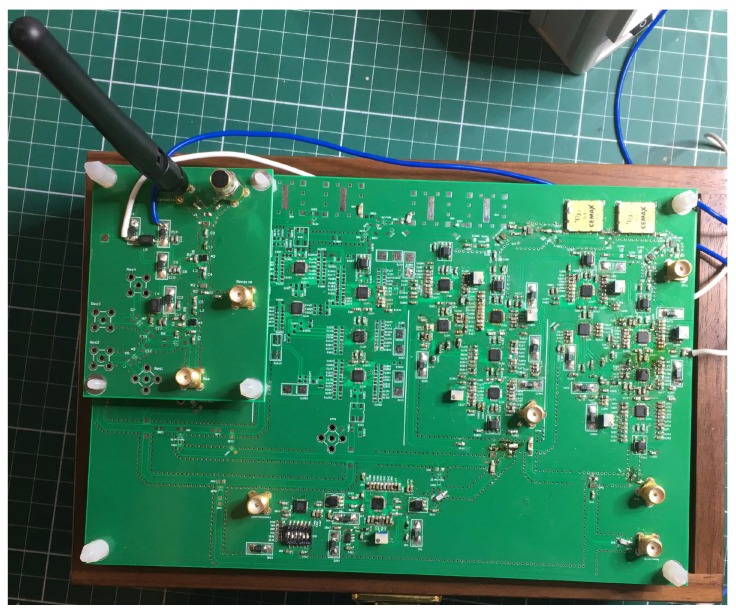
Prototype of the ALMS loop and a part of the receiver.

The prototype of the ALMS loop is designed and fabricated on a Roger 4350B printed circuit board material (Rogers Corporation, Rogers, CT, USA) with all surface mount devices as shown in Figure 5. Since this prototype is implemented to demonstrate the ALMS loop and validate the theoretical results, it is designed in a versatile form so that it can have one to four taps. The amount of delay for each tap can also be changed by cascading multiple delay lines. Therefore, the size of the prototype is 20 cm × 13 cm. In fact, when the ALMS loop is optimized for a specific IBFD system, its size will be much smaller. The dimension of the ALMS loop can be further minimized if it is manufactured in an analog IC.

Detailed descriptions of the components in the ALMS loop are provided as follows. The delay line of the second tap is chosen as $T_{d}=4$ ns using DL4 (RN2 Technologies, Hwaseong-si, Gyeonggi-do, Korea). In each tap, the demodulator ADL5382 and the modulator ADL5373 (Analog Devices, Norwood, MA, USA) are selected for the first and the second multiplier pairs, respectively, because they both have a quadrature structure with differential outputs/inputs for the ease of interfacing. In addition, these components have a very small level of I/Q imbalances, which are less than 0.3 degree in phase imbalance and 0.07 dB in amplitude imbalance. Therefore, according to [24], the level of cancellation will be only degraded by about 0.3 dB (cf. Figure 6 in [24]). Both ADL5382 and ADL5373 require a 0 dBm LO signal, thus $V_{LO}$ is calculated as

(11) $$V_{LO}=\sqrt{2P_{LO}R}=\sqrt{2\times 10^{(0+17-30)/10}}=0.3166\;V,$$

where 17 dB is added to convert the power with a 50 Ohm load to that of an 1 Ohm load. From the ADL5382 datasheet, its conversion gain is 3.5 dB at 2.4 GHz if $P_{LO}=0$ dBm. Since the conversion gain is defined by the ratio between the output power and the input power at the RF port, the dimensional constant $K_{1}$ can be calculated as $K_{1}=0.3166/10^{\left(3.5/20\right)}=0.2116$ V. In case of ADL5373, the output power will be 5 dBm if the baseband input voltage is 1.4 V and $P_{LO}=0$ dBm. Therefore, $K_{2}$ is found as

(12) $$K_{2}=\frac{1.4\times 0.3166}{\sqrt{2\times 10^{(5+17-30)/10}}}=0.7873\;V.$$

All the power splitters/combiners are Anaren PD2328J5050S2HF which have only 0.5 dB insertion loss. The phase shifter MACOM MAPS-010143 is used along with a power combiner to form the subtractor. The variable gain amplifier (VGA Analog Devices ADL5330) is used since its gain can be changed by a controlled voltage which is adjusted by a potentiometer. In addition, the operating frequency of this VGA is from 10 MHz to 3 GHz only, thus the odd harmonics at the outputs of modulators will be attenuated. The ADL5330 at the output of the cancellation circuit is set to have a 22 dB gain. Due to the losses caused by the power combiner (0.5 dB) and the phase shifter (4.5 dB), $G_{O}$ is 17 dB.

Figure 5 also shows a part of the receiver including the power combiner of the subtractor and a LNA MAAL—011078 (MACOM, Lowell, MA, USA), which can provide a 22 dB gain at 2.4 GHz. After the LNA, a power divider is used to provide the loop-back signal and the residual SI signal for measurements. Since the power splitter causes a 0.5 dB loss at each output, the LNA gain in the loop is 21.5 dB, i.e., μ=5.9425. From these parameters and Equation (9), we can determine the loop gain of the prototype as G=20.1, or 26.06 dB.

## 4. Measurement Results

### 4.1. Measurement Setup

**Figure 6:**
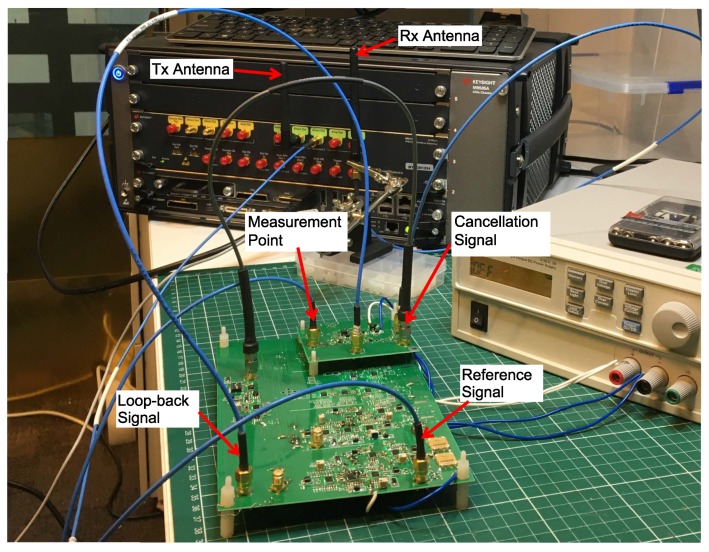
The measurement setup.

In order to evaluate the performance of the prototype, a measurement setup is built as shown in Figure 6.

An arbitrary waveform generator (Keysight M8190A, Santa Rosa, CA, USA) is used as a transmitter. Since one channel of the M8190A has two outputs which can generate the same signals, one of them can be used for the transmitter, and the other is used for the reference signal. In all the tests, the transmitted signal is configured at 2.4 GHz carrier frequency and set at the highest power level of –7.75 dBm. A 2.4 GHz rod antenna is connected directly to one output of M8190A while the other port provides the reference signal for the cancellation circuit. The receive antenna is held on a holder and located at a distance of 75 mm from the transmit antenna. Since the cancellation circuit and the receiver are not electromagnetically shielded, they are located away from the transmitter to reduce the interference to their microstrip lines.

The signal from the receive antenna is connected to one port of the power combiner, while the cancellation signal is connected to the other port. After subtraction, the residual signal is amplified by the LNA and fed into a power splitter, which provides the loop-back signal to the cancellation circuit. The other port of the power splitter is connected to the signal analyzer (Keysight PXA N9030A) for measurements.

### 4.2. Measurement Results

#### 4.2.1. Measurement with Different Bandwidths

**Figure 7:**
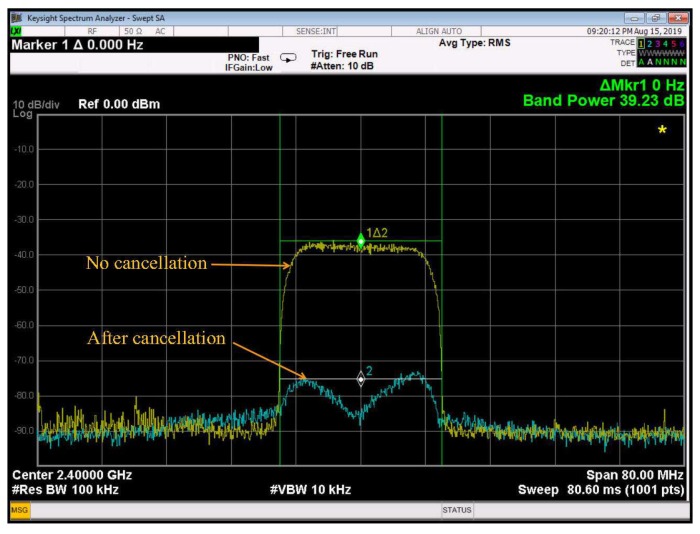
Measurement results for 20 MHz bandwidth.

We evaluate the level of cancellation given by the prototype using the transmitted signals with different bandwidths in the single carrier IBFD system. Transmitted data are modulated with quadrature phase shift keying (QPSK) and then filtered by the RRC pulse shaping filter before up-converted to the RF frequency. The first measurement is conducted with a 20 MHz transmit signal in which the data symbol period is set to $T_{s}=62.5$ ns (i.e., β=0.25). Figure 7 shows the level of cancellation given by the prototype in this case. Marker 1 indicates the difference between the signal power in 20 MHz bandwidth measured at 2.4 GHz of Trace 1 and that of Trace 2. Clearly, a cancellation level of 39.23 dB is achieved by the prototype.

**Figure 8:**
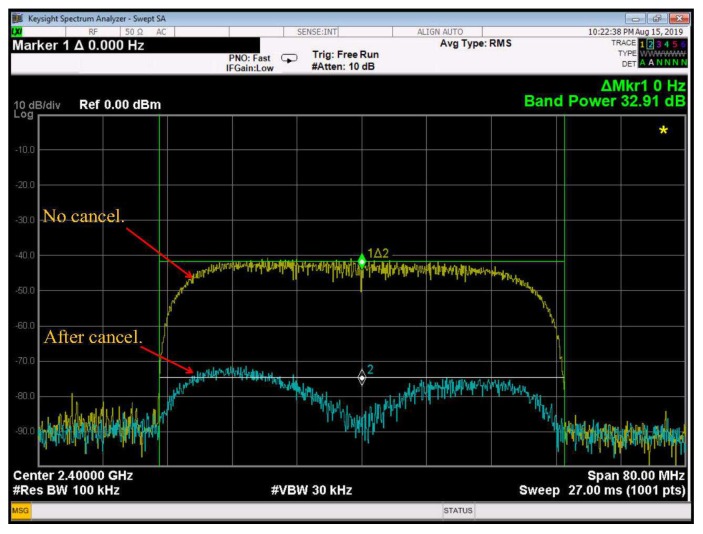
Measurement results for 50 MHz bandwidth.

In the second measurement, the transmitter is set to 50 MHz bandwidth, i.e., $T_{s}=25$ ns and β=0.25. The results of this test are depicted in Figure 8. We can see that a cancellation level of 32.91 dB is achieved in this case.

These experimental results can be used to validate the theoretical results presented in [12,24]. Given the symbol period, the parameter $a^{\prime }$ is determined as 314.06 and 125.625 in the 20 MHz and 50 MHz systems, respectively. Hence, the maximum levels of ISR expected by the ALMS loop calculated from Equation (10) in these two cases are 42.82 dB and 36.53 dB, respectively. Considering I/Q imbalances of the demodulators and modulators, the maximum levels of cancellation will degrade by about 0.3 dB [24], i.e., 42.52 dB and 36.23 dB, respectively. This means that the level of cancellation achieved in the prototype is about 3 dB lower than these analytical maximum levels. This is justifiable because the maximum level of cancellation can only be achieved when the number of taps and tap delay in the loop satisfy the condition that $LT_{d}$ covers the maximum path delay of the SI channel.

#### 4.2.2. Measurement with Different Signal Properties

**Figure 9:**
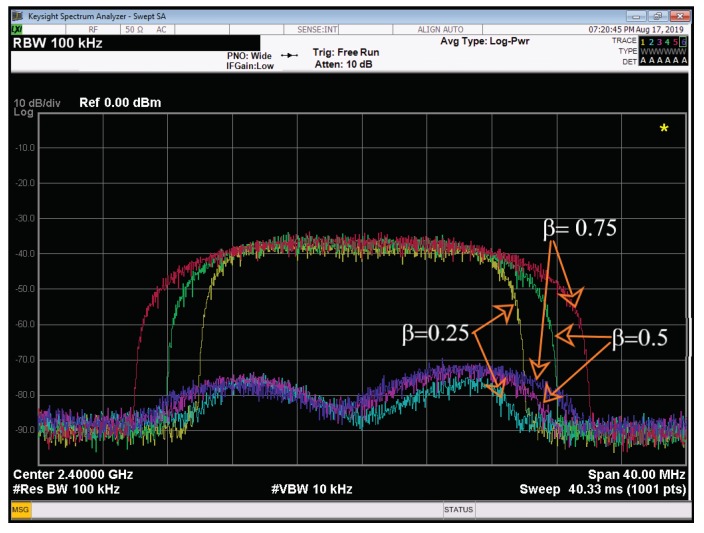
Cancellation performances with different roll-off factors.

In this test, the effect of transmitted signal spectrum on the ALMS loop performance is evaluated. Firstly, the roll-off factor of the pulse shaping filter in the transmitter is configured with different values while the symbol period of the transmit data are fixed at $T_{s}=62.5$ ns. Figure 9 depicts the results of the third test. Measurement results show that the level of cancellation is 39.23 dB, 38.10 dB, and 37.0 dB when the roll-off factor is 0.25, 0.5, and 0.75, respectively. The decrease of ISR with the increased roll-off factor confirms the analyses shown in [12,22].

**Figure 10:**
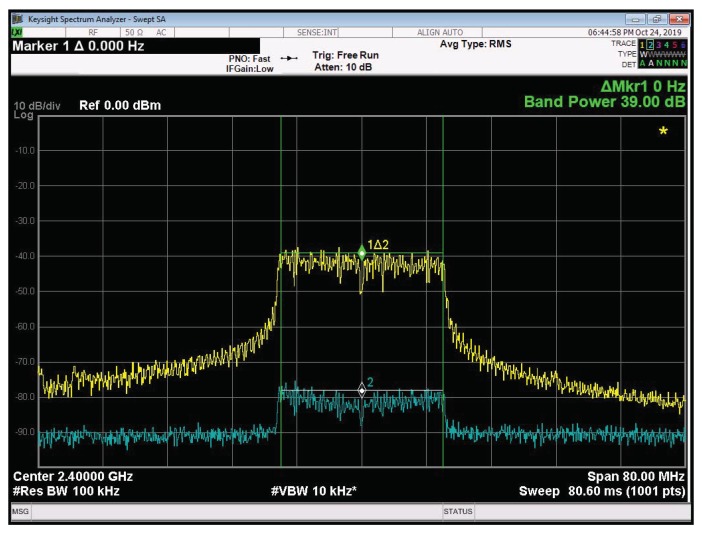
Cancellation performances with the OFDM signal.

In the last test, we consider the performance of the ALMS loop with a multicarrier signal. The transmitter is configured to transmit the OFDM signal based on the IEEE 802.11 a/g format over a bandwidth of 20 MHz. As shown in Figure 10, the level of cancellation in this case is also about 39 dB, which is the same as that in the single carrier system. It means that the ALMS loop works well with both single carrier and multicarrier signaling schemes as concluded in [21,22,24].

## 5. Conclusions

This paper presents a practical structure and implementation of the ALMS loop using off-the-shelf components. The measurement results show that 39.23 dB and 32.91 dB of SI mitigations can be achieved by the prototype for IBFD systems with 20 MHz and 50 MHz bandwidths, respectively. The experimental results with different values of the roll-off factor of the transmit pulse shaping filter also prove that the level of cancellation is affected by the roll-off factor of the pulse shaping filter as analyzed in our previous publications. Finally, the experiment with the IEEE 802.11 a/g OFDM signal proves that the ALMS loop performs well in both single carrier and multi carrier signaling schemes. The proposed ALMS loop implementation structure provides a useful practical solution for IBFD communication applications. Our future works include prototyping a complete IBFD single antenna system with a wider bandwidth and a higher transmit power. We may also investigate the behaviors of the ALMS loop in IBFD MIMO systems with different transmitted baseband signals, such as those proposed in [29,30], and with different wireless network topologies [31,32].

## Figures and Tables

**Table 1:** Comparison of existing multi-tap adaptive filters.

	# of Taps	Delay Line	Tap Weight Control	ISR (dB)	Bandwidth (MHz)
[11]	8	Microstrip trace	FPGA	45	80
[13]	2	Anaren IC	Down converter + Integrator	33	20
[14]	4	Coaxial cable	FPGA	21.6	20
[16]	8	Microstrip trace	FPGA	38	20

**Table 2:** Summary of publications on ALMS loops.

	Signalling	Findings	Methods
[19]	Single carrier	ISR vs. loop gain & β	Cyclostationary & stationary
[21]	Multi carrier	ISR vs. windowing function	Cyclostationary & stationary
[22]	Single & multi carrier	ISRLB vs. β	Cyclostationary
[23]	Chirp signal	Tap delay design for deterministic signal	Stationary
[12]	Single carrier	ISRLB in analog and digital domains	Stationary
[24]	Single & multi carrier	Degradation factor vs. I/Q imbalance	Stationary

## Abbreviations

The following abbreviations are used in this manuscript:

ALMS	Analog Least Mean Square
CSI	Channel State Information
DSP	Digital Signal Processing
FPGA	Field Programmable Gate Array
IBFD	In-Band Full-Duplex
IC	Integrated Circuit
IMT	Impedance Mismatched Terminal
I/Q	In-phase/Quadrature
ISR	Interference Suppression Ratio
LNA	Low-Noise Amplifier
LO	Local Oscillator
LPF	Low-Pass Filter
MIMO	Multiple Input Multiple Output
OFDM	Orthogonal Frequency Division Multiplexing
RC	Resistor–Capacitor
RRC	Root-Raised Cosine
RF	Radio Frequency
SI	Self-Interference
